# Evaluating the effect of educational intervention based on the health belief model on the lifestyle related to premenstrual syndrome and reduction of its symptoms among the first-grade high school girls

**DOI:** 10.1186/s12889-023-15950-y

**Published:** 2023-05-30

**Authors:** Parisa Khalilzadeh, Jamileh Amirzadeh-iranagh, Hamid Reza Khalkhali, Mina Maheri

**Affiliations:** 1 Social Determinants of Health Research Center, Clinical Research Institute, Urmia University of Medical Sciences, Urmia, 5756115198 Iran; 2 Department of Public Health, School of Public Health, Urmia University of Medical Sciences, Urmia, Iran; 3 Patient Safety Research Center, Clinical Research Institute, Urmia University of Medical Sciences, Urmia, Iran; 4 Department of Epidemiology and Biostatistics, School of Medicine, Urmia University of Medical Sciences, Urmia, Iran

**Keywords:** Educational intervention, Health belief model, Premenstrual syndrome, Lifestyle, Girls, First-grade high school

## Abstract

**Introduction:**

A healthy lifestyle can reduce the rate and symptoms of premenstrual syndrome. Thus, the present study evaluates the effect of educational intervention based on the Health Belief Model on the lifestyle related to premenstrual syndrome and reduction of its symptoms among the first-grade high school girls.

**Methods:**

This quasi-experimental study was conducted on 80 first-grade high school girls. They were divided into two intervention and control groups (40 people in each group). The data collection tools included the participants’ demographic information questionnaire, premenstrual symptoms screening tool, and a researcher-made questionnaire based on the constructs of the health belief model about PMS and the behaviors reducing its symptoms. Data were collected in two phases (before and three months after the educational intervention) via WhatsApp. Educational sessions were held in the form of four 45-min sessions for intervention group subjects regarding PMS and the behaviors that reduce its symptoms during one month via WhatsApp.

**Results:**

According to the results of this study, the mean scores of knowledge about PMS and health belief model constructs (including perceived susceptibility), perceived severity, perceived benefits, perceived self-efficacy, cues to action, lifestyle/behaviors that reduce PMS symptoms) and the percentage of people who did not have PMS symptoms or had a mild type of PMS increased significantly after implementing the educational intervention in the intervention group compared to before the intervention and compared to the control group. Also, the perceived barriers construct score PMS decreased significantly.

**Conclusions:**

The health belief model education focused on a healthy lifestyle was effective in reducing PMS symptoms. It is recommended to use the educational intervention designed in this study, along with other health care in schools and during puberty as an easy, low-cost, and effective intervention.

**Supplementary Information:**

The online version contains supplementary material available at 10.1186/s12889-023-15950-y.

## Introduction

Premenstrual syndrome (PMS), which is a common disorder among women of reproductive age, is a set of physical, psychological, mood, and behavioral symptoms that occur periodically in the luteal phase of menstruation (about a or two weeks before the onset of menstruation) [1]. More than 200 symptoms have been attributed to PMS. The most significant of them are depressed mood, anxiety, emotional instability, irritability, reduced interest in usual activities, difficulty in concentration, severe lack of energy and fatigue, changes in appetite and overeating, a disorder in sleep (such as insomnia and oversleeping), a feeling of loss of power or lack of control and physical symptoms (such as headache, heartache, abdominal bloating, breast tenderness, swelling, and weight gain) [2–4]. These symptoms primarily start shortly after ovulation, gradually intensify, and reach a maximum of about 5 days before the onset of menstruation. They are primarily eliminated by the end of menstruation [1, 2]. The severe type of PMS is called premenstrual dysphoric disorder (PMDD) and is one of the psychiatric disorders among women [3]. The prevalence of PMS among adolescent girls is higher than in other age groups. It might disrupt their academic performance, including weakness in doing homework, getting low grades on exams, and increasing absenteeism from schools [1, 4, 5]. It highlights the significance of implementing various interventions to reduce PMS symptoms among students [6].

Several studies have reported the effectiveness of lifestyle modification intervention programs in reducing PMS symptoms, including modification of dietary patterns in the form of low-salt and low-sugar consumption or high fruit and vegetable consumption, physical activity, stress control, and avoiding smoking. They have reported promising results [1, 7]. For example, the study by Ayaz-Alkaya et al. revealed that educational interventions aimed at promoting nutrition, exercise, sleep, and stress management were effective in reducing PMS symptoms [8]. Bakir et al. also showed that education about health-promoting lifestyle aspects such as health responsibility, physical activity, nutrition, spiritual growth, interpersonal relations, and stress management was effective in reducing PMS symptoms [9]. Therefore, the design and implementation of health education interventions focusing on lifestyle modification will be effective in reducing PMS symptoms among students [6, 10].

On the other hand, the effectiveness of health education interventions largely depends on the correct use of theories and models in this science. The use of behavior change theories increases the effectiveness of health education programs and involves individual and environmental factors affecting behavior in health education interventions. Existing theories and models in the science of health education provide a map for the design and implementation of educational interventions, and through them, the effect of educational interventions can be measured and behaviors can be predicted. Theory can also assist planners in determining the most appropriate change strategies, and evaluation outcomes. If positive results are obtained, theory-based training can provide other educators with successful experiences and guide them step by step to achieve their educational goals, prevent trial and error, and lead to save time, cost and other resources [11, 12].

Health Belief Model (HBM) is one of the models in the science of health education that can be used to promote lifestyle and behaviors reducing and preventing PMS symptoms [6, 8]. HBM hypothesizes that adopting a health and preventive behavior depends on a person’s beliefs. Thus, the main focus of the intervention is based on the person’s HBM and trying to change his or her beliefs [6, 11].

According to this model, to adopt a lifestyle and behaviors reducing PMS symptoms, people should first feel threatened by the problem (having PMS or facing its symptoms) (perceived susceptibility). Then, they should perceive this risk and the seriousness of its complications in different physical, psychological, family, social, and economic dimensions (perceived severity) by the positive signals they receive from their surroundings or internal environment (cues to action). Then, they should believe in the usefulness and applicability of the lifestyle and behaviors reducing PMS symptoms (perceived benefits) and consider the factors preventing these behaviors as less expensive than their benefits (perceived barriers). Then, they should feel efficacy to overcome the barriers (self-efficacy), so they finally perform behaviors reducing PMS symptoms [6, 11].

Since every woman’s menstrual cycle starts from adolescence and it is easier to institutionalize healthy health behaviors at this age, and considering the significance of maintaining the health of adolescent girls and the harmful effects of PMS on the various functions of students, especially on their academic performance [4], as well as considering that there is a widespread gender inequality in medical research, which leads to a lack of research on menstrual problems and women’s health issues in general [13, 14], the present study investigates the effect of educational intervention based on the Health Belief Model on the lifestyle related to premenstrual syndrome and reduction of its symptoms among the first-grade high school girls.

## Methods

The present study was a controlled type of quasi-experimental interventional study. It was conducted from the fall of 2019 to the winter of 2021. The statistical population of the study included first-grade high school girls in Urmia, Iran. The inclusion of the study included access to the WhatsApp messaging program and the ability to use this technology, the informed consent of the students and their parents to participate in the study, suffering from moderate to severe PMS based on the result of the initial screening (PSST questionnaire was sent to them via WhatsApp), having regular menstruation including the interval between two periods of 21 to 35 days and the duration of bleeding between 2 and 7 days [15], not using hormonal compounds, drugs or non-drug treatments such as a special diet which affects the PMS, not participating in interventional studies related to PMS during the last 6 months, and the ability to answer questions physically and mentally. The exclusion criteria included suffering from a mild type of PMS and premenstrual dysphoric disorder (PMDD) based on the result of initial screening, incomplete filling out of questionnaires, unwillingness to continue cooperation, the occurrence of unfavorable events (death, illness, etc.), and absence of more than 1 session.

Forty samples were studied in each of the intervention and control groups. Multi-stage cluster sampling method was used for sampling. First, the city of Urmia was divided into 2 districts north and south. Then, two first-grade high schools were selected from each district by the convenience sampling method. Then, one of the schools in each district was randomly selected as the intervention group and the other school as the control group. Then, by referring to the selected schools, one seventh-grade class, one eighth-grade class, and one ninth-grade class were randomly selected from each school. A list of students from each class who met the initial inclusion criteria of the study was prepared (almost 20 students from each class were eligible). Then, the aim of the study was explained to the selected students via a phone call and the samples who were willing to cooperate were asked to complete the PSST electronic questionnaire. The direct link to the PSST electronic form was sent to them via WhatsApp. Then, based on the analysis of the results obtained from this stage, 6-7 students from each seventh-grade, eighth-grade, and ninth-grade students who suffered from moderate to severe PMS based on the PSST questionnaire were selected by the convenience sampling method.

The data collection tool in this study included three sections. The first section of the questionnaire was the demographic information of the participants. The second section was PSST. This questionnaire has 19 questions and two sections. The first section includes 14 questions about mood-behavioral and physical symptoms (for example, have you recently observed signs of anger/irritability in yourself?). The second section, which assesses the effect of these symptoms on people’s lives, includes 5 questions (such as its effect on communication with colleagues and friends). In this questionnaire, 4 criteria have been mentioned for each question, including no, mild, moderate, and severe. They are scored on a Likert scale from 0 to 3.

In PSST, three criteria should be met to diagnose moderate to severe PMS. They include 1- From options 1 to 4, at least one case should be moderate or severe, 2- In addition to the previous case, from options 1 to 14, at least 4 cases should be moderate or severe. 3- There should be a moderate or severe case in the effect of symptoms on life (the last 5 options). To diagnose PMDD, three criteria also should be met. They include 1- From options 1 to 4, at least one case should be severe, 2- In addition to the previous case, from options 1 to 14, at least 4 cases should be moderate or severe. 3- There should be a moderate or severe case in the effect of symptoms on life (the last 5 options) [3, 16]. The subject’s score in each mood-behavioral, physical dimension, and the effect of symptoms on life is obtained from the sum of the answers given to the questions of that dimension. The subject’s total score in this questionnaire is also obtained from the sum of the answers given to all 19 questions.

The range of scores that can be obtained in this questionnaire is between 0 and 57. A higher score indicates the severity of symptoms and problems [16]. Siahbazi et al. translated the Persian version of the PSST in Iran [16]. Its validity and reliability were confirmed. Reliability was evaluated by calculating Cronbach’s alpha coefficient, and its value was obtained at 0.90. Its content validity ratio and content validity index values were obtained at 0.70 and 0.80, respectively, indicating the content validity of this questionnaire. The third section of the researcher-made questionnaire was based on the HBM constructs regarding PMS and behaviors reducing its symptoms. This section includes perceived susceptibility with 9 items (for example, it is more likely that I will get PMS due to stress and pressure of studies), perceived severity with 6 items (for example, having PMS can cause academic drop), perceived benefits with 7 items (for example, having a healthy and appropriate diet can reduce the PMS symptoms), perceived barriers with 5 items (for example, having a healthy lifestyle such as regular exercise is time-consuming),perceived self-efficacy with 7 items (for example, I can do exercise regularly), cues to action with 6 items (for example, holding educational courses by school coaches and health care providers is effective in increasing my motivation to do behaviors that reduce PMS symptoms), behavior with 15 items (for example, I eat foods rich in complex carbohydrates, including fruits, vegetables, and grains), knowledge with 10 items (for example, which of the following cases can increase the risk of premenstrual syndrome? A) Low consumption of sugar and sweets, B) low consumption of foods containing caffeine such as tea and coffee, C) Low consumption of fruits and vegetables was D) I don’t know).

The knowledge questions were in a four-option form including one correct option, two incorrect options, and one I don’t know option. A score of 1 is given to the correct options and a score of zero is given to incorrect and I don’t know options. In general, a higher score indicates more knowledge of the subject. The questions of the constructs of the health belief model are scored on a 5-point Likert scale, ranging from strongly disagree (1), somewhat disagree (2), I have no idea (3), somewhat agree (4), and strongly agree (5). The questions related to behavior (lifestyle) are scored on a 5-point Likert scale, ranging from never (1), rarely (2), sometimes (3), often (4), and always (5). Obtaining a higher score in each construct indicates a good condition of the subject in the studied construct.

Face validity (qualitative and quantitative type) and content validity (quantitative type) methods were used to determine the validity of the researcher-made questionnaire. In the qualitative face validity, 10 students of the target group were interviewed face-to-face and their opinions were obtained and included in the questionnaire [17]. In the quantitative face validity, the impact score was calculated for each question. For this purpose, a panel of experts was used (10 experts in fields related to the research areas, including health education, gynecology, midwifery, and epidemiology). The questions whose impact score was more than 1.5 remained in the questionnaire; otherwise, they were removed [17]. To measure the content validity quantitatively, the mentioned panel was also used. The content validity ratio (using necessity criteria) and content validity index (using relevance, clarity, and simplicity criteria) were calculated. Questions with a content validity ratio of more than 0.62 and a content validity index of more than 0.79 were accepted [17]. Cronbach’s alpha coefficient was used to examine the reliability of the questionnaire. For this purpose, the prepared pilot questionnaire was submitted to 30 people from the target group. After completing the questionnaires, Cronbach’s alpha coefficient was calculated. It was calculated at 0.785 for knowledge, 0.912 for perceived susceptibility, 0.919 for perceived severity, 0.932 for perceived benefits, 0.745 for perceived barriers, 0.932 for perceived self-efficacy, 0.950 for cues to action, and 0.969 for lifestyle 0.969 [17].

In the pre-test stage, the direct link of the demographic information electronic form and the researcher’s questionnaire according to HBM constructs were sent to them via WhatsApp to complete. The questionnaires were self-reported and completed under the guidance of the interviewer. According to the results obtained from the analysis of the data related to the pre-test stage and the HBM constructs, an educational intervention related to the behaviors to reduce PMS symptoms was designed and implemented for the students of the intervention group.

In order to better manage the process of educational intervention and facilitate the participation of all people in group discussions and question-answer, training was held in small groups. In this way, the intervention group members were divided into four 10-individual groups and a WhatsApp group was created for each. The training sessions were held for each group in four 45-min sessions and during 1 month.

Educational intervention was conducted through WhatsApp. In this way, a night before each session, it sent the education content, audio and video files, and assignments to each individual’s private profile and each group’s public space, and they were asked about the time of tomorrow’s session between 8 a.m. to 8 p.m. The training time for 4 groups was adjusted so that they did not interfere with each other. Finally, at the time set for the meeting, the group members discussed and exchanged ideas on WhatsApp online/live about the educational materials by sending text and audio messages in Farsi and reported practical work.

In the current study, according to the educational objectives, four teaching methods were used: lecturing, question-answer, group discussion, demonstration. The lecture method was chosen because it saves time and presents a lot of theoretical content in a short time. The question-answer method was chosen because of encouraging learners to think about the topic of education, evaluating their understanding of the topic of education, and also active participation of learners in the learning process. Group discussion is also one of the effective methods to encourage learners to think and actively participate in learning. Participating in a group discussion, in addition to transferring information, is very useful in improving the attitude of learners. Demonstration is also the best method of learning skills in learners through observation [18, 19]. The details of the training sessions are presented in the Appendix.

The control group received the routine health education of the schools, which had the same topics as the educational content of the intervention group. They received it through their school health coach in the lecture and non-theory based method [20]. Of course, to observe the ethical considerations, the designed educational package with a brief explanation was also submitted to the control group at the end of the study. Three months after the end of the training intervention, to perform the post-test, the questionnaires sent in the pre-test stage and the PSST questionnaire were sent to the intervention and control groups via WhatsApp to complete. A phone call was made with the students every two weeks to review the educational materials and remind them during the three months of waiting.

All the steps related to this research, including completing questionnaires, holding online/live training sessions and sending text, audio and video messages were taken via WhatsApp owing to the spread of the Covid-19 disease and the closure of schools.

The data were analyzed in SPSS 16 software using descriptive statistics (mean, standard deviation, percentage, and frequency) and analytical statistics including Kolmogorov-Smironov (to examine the normality of the data), Chi-square, Fisher’s exact test, independent t-test, U Mann Whitney, Paired t-test, McNemar, and analysis of covariance. Effect size classification was considered for analysis of covariance based on Cohen’s guidelines (weak = 0.01, medium = 0.06, and high = 0.14) [21]. The results were considered statistically significant at the *p*<0.05 level.

## Results

According to the results, no statistically significant difference was observed between the two intervention and control groups regarding the variables of age, parents’ age, family size, education level, parents’ education, economic status, and ethnicity before the educational intervention (*p* < 0.05) (Table 1).

**Table 1:** Comparison of the demographic characteristics of the two intervention and control groups in the stage before the educational intervention

Qualitative variable		Group				*p*
		Intervention		Control		
		Number	Percent	Number	Percent	
Grade	Seventh grade	13	32.5	13	32.5	^a^1
	Eighth grade	13	32.5	13	32.5	
	Ninth grade	14	35.0	14	35	
Mother’s education	Illiterate	2	5.0	2	5.0	^b^0.761
	Primary education	7	17.5	5	12.5	
	Intermediate Education	6	15	5	12.5	
	High school and Diploma	13	32.5	15	37.5	
	Associate Degree	6	15	5	12.5	
	Bachelor degree	6	15	5	12.5	
	Master's degree	0	0.0	3	7.5	
Father’s education	Illiterate	0	0.0	0	0.0	^b^0.544
	Primary education	1	2.5	1	2.5	
	Intermediate Education	8	20.0	4	10.0	
	High school and Diploma	9	22.5	12	30.0	
	Associate Degree	6	15.0	11	27.5	
	Bachelor degree	14	35.0	10	25.0	
	Master’s degree	2	5.0	2	5.0	
Economic status	Excellent	3	7.5	4	10.0	^b^0.611
	Good	28	70.0	23	57.5	
	Average	9	22.5	13	32.5	
Ethnicity	Kurd	13	32.5	11	27.5	^a^0.808
	Turkish	27	67.5	29	72.5	
Quantitative variable		Mean	Standard deviation	Mean	Standard deviation	*p*
Student age (years)		14.13	0.883	14.15	1.100	^a^0.792
Mother’s age (years)		63.42	3.972	44.10	5.486	^d^0.173
Father’s age (years)		47.45	5.267	48.53	5.272	^d^0.364
Family size		4.03	0.660	4.05	0.737	^c^0.762

^a^Chi-squared test

^b^Fisher’s exact test

^c^Mann-Whitney U test

^d^Independent t-test

According to the results of the present study, there was no statistically significant difference between the two intervention and control groups regarding the mean scores of knowledge and constructs of perceived susceptibility, perceived severity, perceived benefits, perceived barriers, perceived self-efficacy, cues to action, and lifestyle (behaviors) related to PMS and the reduction of its symptoms before the educational intervention (*p* < 0.05). However, these differences were statistically significant after the educational intervention (*p* < 0.05) (Table 2).

**Table 2:** Comparison of the mean scores of the constructs of the health belief model regarding PMS and the reduction of symptoms caused by it before and three months after the educational intervention in two intervention and control groups

Variable	Research stage	Mean ± Standard deviation		*p*-between	*η*^*2*^*p*	**p-between-adjusted	*η*^*2*^*p*
		Intervention group	Control group				
knowledge	Pre-test	1.48 ± 3.42	2.27 ± 3.82	^a^.355			
	Post-test	1.26 ± 5.70	2.21 ± 3.95	0.001 > ^b*^	0.620	0.001 > ^b^	0.660
	p-within	0.001 > ^c^	^c^ 0.058				
Perceived sensitivity	Pre-test	4.12 ± 28.80	4.38 ± 30.05	^a^ 0.193			
	Post-test	3.43 ± 31.85	5.29 ± 29.62	0.001 > ^b*^	0.328	0.001 > ^b^	0.328
	p-within	0.001 > ^c^	0.379^c^				
Perceived severity	Pre-test	3.32 ± 21.62	2.30 ± 21.95	^a^0.613			
	Post-test	2.40 ± 23.05	2.16 ± 22.15	0.001 > ^b*^	0.267	0.001 > ^b^	0.288
	p-within	0.001 > ^c^	0.044^c^				
Perceived benefits	Pre-test	3.95 ± 23.15	3.78 ± 24.00	^a^ 0.330			
	Post-test	2.82 ± 25.30	3.65 ± 24.12	0.001 > ^b*^	0.410	0.001 > ^b^	0.438
	p-within	0.001 > ^c^	0.230^c^				
Perceived barriers	Pre-test	3.66 ± 15.90	4.10 ± 16.20	^a^ 0.731			
	Post-test	3.21 ± 12.92	4.04 ± 16.02	0.001 > ^b*^	0.371	0.001 > ^b^	0.415
	p-within	0.001 > ^c^	0.147^c^				
Perceived self-efficacy	Pre-test	3.86 ± 26.82	4.17 ± 26.92	^a^ 0.912			
	Post-test	2.58 ± 28.90	4.15 ± 27.02	0.001 > ^b*^	0.438	0.001 > ^b^	0.460
	p-within	0.001 > ^c^	0.210^c^				
Cues to action	Pre-test	3.17 ± 22.40	2.80 ± 22.07	^a^ 0.629			
	Post-test	2.58 ± 24.00	2.84 ± 22.10	0.001 > ^b*^	0.342	0.001 > ^b^	0.359
	p-within	0.001 > ^c^	0.323^c^				
Lifestyle	Pre-test	8.51 ± 47.75	5.23 ± 48.67	^a^ 0.560			
	Post-test	7.61 ± 51.87	5.21 ± 48.87	0.001 > ^b*^	0.360	0.001 > ^b^	0.365
	p-within	0.001 > ^c^	0.448^c^				

^a^Independent T-test

^b^ANCOVA

^c^Paired T-test

η^2^p, Partial Eta Squared and interpreted following the Cohen guidelines for small effect sizes (partial eta^2^ = 0.01), moderate effect sizes (partial eta^2^ = 0.06), and large effect sizes (partial eta^2^ = 0.14) (15)

^*^Result of ANCOVA adjusted for pre-test score

^**^Result of ANCOVA adjusted for pre-test score, age, educational level, parent's education, economic status and ethnicity

Also, intra-group comparisons revealed a statistically significant difference in the intervention group regarding the mean scores of all studied constructs before and after the educational intervention (*p* < 0.05). However, these differences were not statistically significant in the control group (*p* < 0.05) except for the construct of perceived severity (Table 2).

According to the results of this study, there was no statistically significant difference between the two intervention and control groups regarding the mean score of premenstrual syndrome before the educational intervention (*p* = 0.161). However, the difference was statistically significant after the educational intervention (*p* < 0.001). Also, the intra-group comparison revealed a statistically significant difference between the mean scores of premenstrual syndrome in the intervention group before and after the educational intervention (*p* < 0.001). However, this difference was not statistically significant in the control group (*p* = 0.448) (Table 3).

**Table 3:** Comparison of the mean score of premenstrual syndrome before and three months after the educational intervention in two intervention and control groups

Variable	Research stage	Mean ± Standard deviation		*p*-between	*η*^*2*^*p*	*p*-between-adjusted	*η*^*2*^*p*
		Intervention group	Control group				
Premenstrual syndrome	Pre-test	2.44 ± 24.90	2.29 ± 24.15	^a^ 0.161			
	Post-test	3.33 ± 21.97	2.90 ± 24.37	0.001 > ^b*^	0.333	0.001 > ^b^	0.386
	p-within	0.001 > ^c^	^c^0.448				

^a^Independent T-test

^b^ANCOVA

^c^Paired T-test

η^2^p, Partial Eta Squared and interpreted following the Cohen guidelines for small effect sizes (partial eta^2^ = 0.01), moderate effect sizes (partial eta^2^ = 0.06), and large effect sizes (partial eta^2^ = 0.14) (15)

^*^Result of ANCOVA adjusted for pre-test score

^**^Result of ANCOVA adjusted for pre-test score, age, educational level, parent's education, economic status and ethnicity

According to the results of this study, there was no statistically significant difference between the two intervention and control groups regarding the frequency of premenstrual syndrome severity before the educational intervention (*p* = 1). However, this difference was statistically significant after the educational intervention (*p* = 0.002). The intra-group comparison revealed a statistically significant difference between the two stages before and after the educational intervention regarding the frequency of premenstrual syndrome severity in the intervention group (*p* < 0.001). However, this difference was not statistically significant in the control group (*p* = 0.414) (Table 4).

**Table 4:** Comparison of the frequency of severity of premenstrual syndrome before and three months after the educational intervention in two intervention and control groups

Variable	Research stage	Intervention group			Control group			*p*-between
		Absence of PMS/ Mild	Moderate to severe PMS	PMDD	Absence of PMS/ Mild	Moderate to severe PMS	PMDD	
Premenstrual syndrome	before the intervention	0 (0)	40 (100)	0 (0)	0 (0)	40 (100)	0 (0)	^a^1
	after the intervention	13 (32.5)	26 (65.0)	1 (2.5)	2 (5.0)	34 (85.0)	4 (10)	^a^0.002
	p-within	0.001 > ^b^			0.414^b^			

^a^Chi-squared test

^b^McNemar’s test

## Discussion

The results revealed the effectiveness of the designed educational intervention in enhancing the level of knowledge of students in the intervention group about PMS and the lifestyle/behaviors that reduce its symptoms. Other studies have also proven the effectiveness of educational interventions based on HBM in enhancing the level of people’s health knowledge. For example, the study by Khalilipour et al. reported that the mean score of knowledge about PMS significantly increased after an educational intervention based on HBM in the intervention group compared to before the intervention and compared to the control group [6].

The study by Nooshin et al. also revealed that HBM-based education significantly increases students’ knowledge about healthy lifestyles. One of the significant methods in creating correct health knowledge is designing and implementing proper and principled education. For example, education by school health educators can be very helpful in enhancing adolescent girls’ knowledge about the issues related to puberty. Health education based on educational models and theories is also effective in enhancing knowledge, changing attitudes, and adopting healthy behaviors [22].

The results also revealed that the implemented educational intervention was effective in increasing the perceived susceptibility of girl students to the possibility of PMS. Since the perceived susceptibility has a strong cognitive component and depends on the individual’s knowledge awareness to some extent [11], it can be concluded that enhancing the level of knowledge and information of the intervention group about complications and problems caused by premenstrual syndrome increased their perceived susceptibility. Hence, the intervention group, compared to the control group, considered themselves more susceptible to PMS. Additionally, the improvement in the perceived susceptibility is related to the strategies that have been used in the present study, including providing statistics on the prevalence of PMS and stating the risk and predisposing factors of PMS for the girl students [11]. Some of these predisposing factors include stress, an unhealthy diet, and lack of exercise.

The results indicate the effectiveness of the implemented educational intervention in increasing the perceived severity of the female students regarding the symptoms and complications of PMS. Thus, it is recommended to include the strategies that improve the perceived severity in all interventions designed and implemented to improve the lifestyle related to the reduction of PMS symptoms among the girl students (such as stating the physical and psychological complications of PMS through lectures or sheets containing facts, showing the intensity and seriousness of the case, for example, telling a story about a student who suffered academic failure owing to lack of control of PMS symptoms) [11]. People’s perception and assessment of risk is the primary element of HBM. This construct should be strengthened by parents, school health educators, and health educators as a significant factor in the formation of behavior in which there is weakness [23] since low perceived severity is one of the main barriers to adopting healthy and preventive behaviors [24].

The educational intervention designed in the present study has been effective in increasing the perceived benefits related to lifestyle/behaviors that reduce PMS symptoms. In order to improve the construct of perceived benefits resulting from performing a specific behavior, it is necessary to emphasize and focus on benefits that are important and valuable from the point of view of the target group. Therefore, in this study, in order to strengthen the perceived benefits related to lifestyle/behaviors that reduce PMS symptoms, benefits were always expressed and discussed, which were important and valuable from the point of view of teenage school girls. Examples of these perceived benefits included reduced PMS symptoms, weight management, and improved academic performance, interpersonal relationships, and overall health. Also, another strategy that can lead to the improvement of the construct of perceived benefits is specifying the exact method of performing the desired behavior. For this reason, in this study, the target group was taught the exact method of performing behaviors that lead to the reduction of PMS symptoms. For example, they were taught to do aerobic exercise at least 3 days a week and for at least 30 min each time, and to eat more and smaller meals [11].

In the present study, following the implementation of the educational intervention, perceived barriers related to lifestyle/behaviors that reduce PMS symptoms were reduced in the intervention group. It should be noted that given to the nature of perceived barriers, its change required more time, energy and effort compared to other constructs of the HBM. Therefore, it is suggested to pay attention to this matter in similar studies. Among the strategies that can lead to the reduction of perceived barriers related to a specific behavior include reassuring the target group that performing the desired behavior will impose little cost, for example, stating that exercising will only take 30 min of your time per day. Also, correcting misperceptions related to the desired behavior can also reduce the perceived barriers, for example, students may think that having adequate sleep is not possible due to the special conditions of their studies. In this case, teaching the skill of planning to do things can help to resolve this misunderstanding. Also, providing financial and non-financial incentives will be effective in reducing perceived barriers [11]. All these strategies were used in this study. Also, educational strategies related to increasing the construct of perceived severity, in addition to improving this construct, can indirectly reduce perceived barrier [25]. High self-efficacy reduces the perceived barriers related to healthy behaviors [26].

The results also revealed the effectiveness of the designed educational intervention in increasing the perceived self-efficacy of students in the intervention group to perform lifestyle/behaviors that reduce PMS symptoms. Some of the strategies used in the present study to improve self-efficacy were teaching behaviors that help reduce PMS symptoms in the form of small, simple, and performable steps (such as teaching home exercises in the form of small, simple, and performable steps to students), using believable role models (such as the introduction of students who have reduced and controlled their PMS symptoms by performing healthy behaviors), using persuasion and verbal reinforcement, and teaching relaxation techniques to reduce stress [11, 27]. Using these strategies should be included in other educational programs aimed at promoting behaviors that reduce PMS symptoms.

In the present study, the external stimuli used as a cue to action to facilitate and accelerate the decision of the students in the intervention group to do lifestyle/behaviors that reduce PMS symptoms included sending educational packages including audio-visual clips, posters, pamphlets, and educational booklets about PMS and behaviors reducing its symptoms, sending a summary of educational content related to lifestyle/behaviors that reduce PMS symptoms to at least one member of the family, teacher, and health care provider of schools in the intervention group and asking them to encourage their student to do lifestyle/behaviors that reduce PMS symptoms and make phone calls once every two weeks during the 3 months of waiting after the end of the educational intervention to review the educational materials. It is recommended to include all these stimuli in educational programs aimed at promoting behaviors that reduce PMS symptoms.

The results of the present study revealed that the educational intervention designed and implemented based on HBM constructs was effective in promoting lifestyle/behaviors that reduce PMS symptoms among girl students. Consistent with the results of the present study, Kamili and Jalili’s study showed that the score of PMS preventive behaviors in the intervention group increased significantly after implementing educational intervention based on HBM [6].

This study revealed that the mean PMS score was significantly reduced, and the percentage of people without PMS symptoms or suffering from mild PMS increased significantly after implementing the educational intervention in the intervention group compared to before the intervention and compared to the control group. This result suggests that the designed and implemented education based on HBM constructs focused on a healthy lifestyle and a set of strategies, techniques, methods, contents, materials and educational tools, and other educational resources used in the present study was effective in improving and reducing PMS symptoms. Other studies have also confirmed the effectiveness of educational interventions related to a healthy lifestyle in reducing PMS symptoms. For example, in a study entitled "Effect of educational program on premenstrual syndrome in adolescent school girls", a significant reduction was observed in the severity of PMS symptoms after teaching a healthy lifestyle (including stress management, healthy diet, and physical activity and exercise) Ramya et al. [10].

## Conclusion

Based on the results and the rate of progress and changes observed, it can be concluded that the educational intervention based on HBM focused on a healthy lifestyle is effective in reducing PMS symptoms. Given the significance of maintaining the health of adolescent girls and the destructive effects of PMS on their academic performance, as well as considering the positive results obtained from the present study, it is recommended to use the educational intervention designed in this study, along with other health care programs in schools and during puberty as an easy, low-cost, and effective intervention, since the implementation of such educational-supportive interventions can reduce PMS symptoms and improve the quality of life by promoting the healthy lifestyle of adolescent girls. The knowledge, expertise, experience, and capabilities of health education and promotion experts can be used in different environments (including health centers and schools) to achieve this goal.

## Supplementary Information

**Additional file 1.** Details of the training sessions.

## Data Availability

The datasets used and/or analyzed during the current study are available from the corresponding author on reasonable request.

## Abbreviations

PMS: Premenstrual syndrome
PMDD: Premenstrual dysphoric disorder
PSST: Premenstrual symptoms screening tool
HBM: Health belief model
SPSS: Statistical package for the Social Sciences
COVID-19: Coronavirus disease 2019
etc: Et cetera
et al: Et alia
η^2^p: Partial Eta squared
ANCOVA: Analysis of covariance
