# Supplementary material for: Evaluating the effect of educational intervention based on the health belief model on the lifestyle related to premenstrual syndrome and reduction of its symptoms among the first-grade high school girls
Source: BMC Public Health. 2023 May 30;23:1001. doi: 10.1186/s12889-023-15950-y (PMC10227400; doi:10.1186/s12889-023-15950-y)
Supplement: Supplementary file 1 — Additional file 1. Details of the training sessions. [file 12889_2023_15950_MOESM1_ESM.docx]

Details of the training sessions

| **Session** | **Intervened construct** | **Goal** | **Outlines** | **Educational methods** | **Educational materials and media** | **Education time** | **Assignments given at the end of the education** | **Evaluation method** |
| --- | --- | --- | --- | --- | --- | --- | --- | --- |
| 1 | Knowledge | Enhancing the knowledge of the intervention group about premenstrual syndrome | 1- Introduction and communication  2- Definition of premenstrual syndrome:  3- Stating the signs and symptoms of premenstrual syndrome  4- Stating the cause of the premenstrual syndrome  5- Stating the relationship between lifestyle and premenstrual syndrome, including the effect of nutrition on premenstrual syndrome (high consumption of high-sugar foods, high-fat foods, foods containing caffeine such as tea and coffee, low consumption of fruits and vegetables and water, lack of stress management, insufficient and poor quality sleep, lack of physical activity, smoking and alcohol consumption) | Lecture, and Question-answer | Mobile phones, tablets, audio-video clips, photos, educational booklets, health message posters, and pamphlets | 45 minutes | Studying the slides and watching the provided educational video, reviewing and preparing a report on the type and level of physical activity and exercise, diet, stress management, sleep pattern, and smoking and alcohol consumption. | Performing pre-test and post-test using knowledge assessment questionnaire |
| 2 | Perceived susceptibility and perceived severity | - Increasing the perceived susceptibility of the intervention group to the possibility of premenstrual syndrome  -Increasing the perceived severity of the intervention group regarding the symptoms and complications of premenstrual syndrome | 1- Providing statistics on the prevalence of premenstrual syndrome (statistics related to the world, Iran, and adolescent girls)  2- Stating the risk factors of premenstrual syndrome, including age, genetics, psychological factors, nutritional factors, etc.  3- Stating the complications and negative consequences caused by premenstrual syndrome on social relationships, occupational and academic activities, body, mind, etc. | Lecture, and Question-answer | Mobile phones, tablets, audio-video clips, photos, educational booklets, health message posters, and pamphlets | 45 minutes | Studying the slides and watching the provided educational video - preparing and presenting a report of the risk factors in the student that can be modified by changing the lifestyle.  - The student should write down the symptoms of premenstrual syndrome that he or she experiences and share them with his or her classmates in the next session. | Performing a pre-test and post-test using a questionnaire assessing perceived susceptibility and perceived severity |
| 3 | Perceived benefits and perceived barriers | - Increasing perceived benefits due to adherence to the behaviors reducing premenstrual syndrome symptoms  -Reducing the perceived barriers to adhering to the behaviors reducing the symptoms of premenstrual syndrome | 1- Stating the benefits of having a healthy diet, regular physical activity, stress management, and adequate and high-quality sleep in reducing the symptoms of premenstrual syndrome.  2- Stating the benefits of avoiding smoking and alcoholic drinks in reducing the symptoms of premenstrual syndrome  3- Stating the barriers to having a healthy diet, regular physical activity, stress management, and sufficient, and high-quality sleep  4- Stating the barriers to avoiding the consumption of tobacco, and alcoholic drinks | Group discussion, and Question-answer | Mobile phones, tablets, audio-video clips, photos, educational booklets, health message posters, and pamphlets | 45 minutes | The students were asked to provide a list of benefits that they will get by following a healthy lifestyle in addition to the discussed items.  - The students were asked to list the real and subjective barriers caused by healthy behavior in addition to the discussed items. | Performing a pre-test and post-test using a questionnaire assessing perceived benefits and perceived barriers |
| 4 | Perceived barriers and perceived self-efficacy | -Reducing the perceived barriers to adhere to the behaviors reducing the symptoms of premenstrual syndrome  -Increasing the perceived self-efficacy to adhere to the behaviors reducing premenstrual syndrome symptoms | 1- Resolving cognitive misunderstandings related to healthy eating, regular physical activity, stress management, sufficient and high-quality sleep, smoking, and alcoholic drinks  2- Teaching behaviors that reduce the symptoms of premenstrual syndrome in the form of practical and performable small steps  3- Performing the necessary follow-ups to repeat and continue the behaviors suggested by the teacher  4- Sending reinforcing messages with the keyword "I can" | Demonstration, Question-answer, and Group discussion | Mobile phones, tablets, audio-visual clip, educational booklet, message-reinforcing posters | 45 minutes | - The students were asked to provide solutions to overcome the barriers  Students were asked to:  - Do aerobic exercises including fast walking, swimming, and cycling at least 3 days a week and for at least 30 minutes each time.  - Limit the use of sugar and sweets, high-fat foods, caffeine-containing foods such as tea and coffee, and salt and salty foods.  - Use the foods rich in complex carbohydrates, including fruits, vegetables, and grains, and foods rich in calcium, such as milk, yogurt, and cheese.  -Drink at least six glasses of water daily.  -Eat more and smaller meals  - Use herbal teas such as ginger and chamomile  - Stay away from stressful situations  -Spend time relaxing every day.  - Have at least 8 hours of regular night sleep  -Avoid smoking and being in environments contaminated by the smoke of smokers. | Conducting a pre-test and post-test using a questionnaire assessing perceived barriers and perceived self-efficacy |
| - | Cues to action | Providing cues to action to adhere to the behaviors reducing the symptoms of premenstrual syndrome | 1- Sending an educational package including audio-video clips, posters, pamphlets, and educational booklets about premenstrual syndrome, and the behaviors reducing its symptoms  2- Holding educational sessions about premenstrual syndrome and behaviors reducing symptoms caused by it  3- Sending a summary of the educational content related to the lifestyle that reduces the symptoms of PMS to at least one family member of the subjects, as well as the teacher and health care providers of the schools of the intervention group and asking them to encourage their students to do behaviors that reduce the symptoms of premenstrual syndrome  4- Phone call once every two weeks during the 3 months of waiting after the end of the educational intervention to review the educational materials and remind them | Lecture, Demonstration, Question-answer, and Group discussion | Mobile phones, tablets, audio-video clips, photos, educational booklets, health message posters, and pamphlets | During the educational intervention and during the 3 months of waiting after the end of the educational intervention | The students were asked to pay attention to the stimuli that facilitate and accelerate their decision to perform behaviors that help to reduce the symptoms of premenstrual syndrome. | Performing a pre-test and post-test using a questionnaire to cues to action |
